# Comparative gene expression pattern of immune-related genes using dual-color RT-MLPA in the lesions of cutaneous leishmaniasis caused by *L. major* and *L. tropica*

**DOI:** 10.1371/journal.pntd.0012812

**Published:** 2025-03-18

**Authors:** Nasrin Masoudzadeh, Mouad Ait Kbaich, Suzanne van Veen, Björn Andersson, Marielle C. Haks, Josefine Persson, Vahid Mashayekhi Goyonlo, Shima Hadifar, Reza Erfanian Salim, Idris Mhaidi, Myriam Riyad, Khadija Akarid, Ali M. Harandi, Tom HM Ottenhoff, Meryem Lemrani, Sima Rafati

**Affiliations:** 1 Department of Immunotherapy and Leishmania Vaccine Research, Pasteur Institute of Iran, Tehran, Iran; 2 Laboratory of Parasitology and Vector-Borne-Diseases, Institut Pasteur du Maroc, Casablanca, Morocco; 3 Molecular Genetics and Immunophysiopathology Research Team, Health and Environment Laboratory, Aïn Chock Faculty of Sciences, University of Hassan II, Casablanca, Morocco; 4 Department of Infectious Diseases, Leiden University Medical Center, Leiden, The Netherlands; 5 Bioinformatics Core Facility, Sahlgrenska Academy, University of Gothenburg, Gothenburg, Sweden; 6 Department of Microbiology and Immunology, Institute of Biomedicine, Sahlgrenska Academy, University of Gothenburg, Gothenburg, Sweden; 7 Cutaneous Leishmaniasis Research Center, Mashhad University of Medical Sciences, Mashhad, Iran; 8 Noor Eye Hospital, Tehran, Iran; 9 Laboratory of Cellular and Molecular Pathology, Research Team on Immunopathology of Infectious and Systemic Diseases, Medicine and Pharmacy Faculty, University of Hassan II, Casablanca, Morocco; Institute of Postgraduate Medical Education and Research, INDIA

## Abstract

Cutaneous leishmaniasis (CL) is the most prevalent type of leishmaniasis disease and causes skin lesions, mainly ulcers, on exposed parts of the body. The Americas, Mediterranean basin, Middle East, and Central Asia account for approximately 95% of all CL cases. *Leishmania* (*L.*) *major* and *L. tropica* are the most significant species causing CL. A better understanding of the molecular mechanisms of CL caused by *Leishmania* parasite species in patients’ skin lesions may help inform intervention approaches. Using dual-color reverse transcriptase multiplex ligation-dependent probe amplification (dcRT-MLPA), we evaluated the expression of 144 host immune-related genes in lesions from CL patients infected with two *Leishmania* species, *L. major* and *L. tropica*, in Morocco and Iran, respectively. Distinct gene expression patterns were identified in the lesions of patients infected with *L. major* and *L. tropica*. The results revealed that *L. tropica*-infected patients had rather more significant gene expression than *L. major*-infected patients relative to healthy volunteers. However, CD14 and IFI6 (interferon alpha inducible protein 6), were two common genes expressed in the lesions of patients infected with *L. major* and *L. tropica*. Our analysis revealed that gene expression changes related to the IFN signaling pathway were significant in both lesion groups. This research advances our understanding of the host immune response to zoonotic and anthroponotic leishmaniasis and shows immune transcript signatures in the skin lesions of CL patients infected with *L. major* and *L. tropica*. These findings can inform further investigation into the processes underpinning immunity and immunopathology of CL caused by *L. major* and *L. tropica*.

## Author summary

Cutaneous leishmaniasis (CL) is a significant global public health challenge. The prevailing forms of CL in Morocco and Iran are caused by *Leishmania major* (*L. major*) and *L. tropica*. *L. major* is transmitted following the bite of infected phlebotomine sand flies from animals to humans, while *L. tropica* spreads directly between people. Currently, there is a lack of sufficient transcriptome studies in the lesions of CL patients induced by different *Leishmania* species. This study focused on transcriptome analysis of the immune responses in Moroccan patients infected with *L. major* and Iranian patients infected with *L. tropica*. Using dual-color RT-MLPA, we examined the expression of genes related to innate, adaptive, and inflammatory responses in both patient groups. There are significant changes in the expression of several inflammatory genes in each group. Notably, patients with *L. tropica* lesions exhibited a higher number of differentially expressed genes compared to those with *L. major*, indicating a stronger inflammatory response associated with *L. tropica* infection. It’s crucial to highlight that genes involved in the IFN signaling pathway were significantly altered in the skin lesions of both groups. Understanding these differences will be pivotal in identifying potential targets for therapeutic intervention to control the disease effectively.

## 1. Introduction

Cutaneous leishmaniasis (CL) is a multifaceted disease that continues to be a global public health issue, with an estimated 1 million new cases reported each year. CL is the most frequent type of leishmaniasis in more than 90 countries and is defined mainly by ulcerative skin lesions, with 95% of cases happening in South and Central America, the Mediterranean Basin, West Africa and spreading across the Middle East to Central Asia [1–3]. Among them, CL remains notable concern in Morocco within the Mediterranean Basin and in Iran in the West of Asia, with high infection rates reported in certain regions of both countries [4,5]. The most common variants of CL in both nations are caused by *Leishmania major* (*L. major*), which causes the zoonotic form of CL, and *L. tropica*, which causes the anthroponotic type of CL disease [4].

Despite a great deal of information available about the immune responses to CL in mouse models, understanding of the local immune responses to CL in humans is limited [6–8]. Recent studies by us and others on transcriptomics analysis of CL lesions have contributed to the identification of the gene expression profiles linked to immunopathogenesis in host and pathogen response [9,10]. We recently reported the whole genome transcriptome of CL lesions using RNA sequencing and identified the molecular markers of *L. tropica*-infected patients with various clinical presentations. In this study, we identified shared and unique immunological pathways and inflammatory proteins in the skin lesion of CL patients [11]. Despite this progress, there are still few studies that compare the transcriptional markers of CL lesions induced by different *Leishmania* species [12]. Further transcriptome analysis of the immune responses of the two patient groups infected with distinct *Leishmania* parasites may help to inform the rational design of diagnostics and innovative interventions against CL.

In the present study, we employed a dual-color reverse-transcription multiplex ligation-dependent probe amplification (dcRT-MLPA) method and a systems biology approach to quantify and compare the expression of 144 genes in skin biopsy samples obtained from CL patients infected with *L. major* (Morocco) or *L. tropica* (Iran) in comparison to samples from healthy volunteers. Results showed significant changes in the expression of several inflammatory genes in each patient group when compared with the healthy volunteers. Further, genes involved in the IFN signaling pathway were significantly changed in skin lesions of CL patients infected with *L. major* or *L. tropica*. These results provide new insights into the comparative local immune response to CL induced by *L. major* and *L. tropica* relative to the healthy volunteers.

## 2. Materials and methods

### 2.1. Ethics statement

All adult participants in the study provided signed informed consent for Moroccan patients. Written consent for the inclusion of young children was obtained from parents or guardians. The study and methods were authorized by the Ethics Committee for Biomedical Research (CERB) of the Faculty of Medicine and Pharmacy, Rabat, Morocco (IORG 0006594, approved 16 April 2019).

For Iranian CL patients infected with *L. tropica* and healthy volunteers, the study was conducted according to the principles of the Declaration of Helsinki and under local ethical guidelines. Ethics Committee of Biomedical Researches at Pasteur Institute of Iran (IR.PII. REC.1398.044) approved all methods and consent forms. All CL patients and healthy volunteers received information about the investigation, and signed informed consent were obtained prior to collection of samples.

### 2.2. Study design for CL patients infected with *L. major* and *L. tropica*

Biopsies from fourteen Moroccan patients with skin lesions suggestive of CL were taken from three hospitals: (i) Department of Dermatology - Ibn Rochd Hospital, Casablanca; (ii) Bougafer Hospital in Ouarzazate; (iii) Tinghir Hospital Center. The enrolled patients had not received any prior anti-leishmanial treatment, and their lesions were in the acute phase, with a maximum duration of 4 months. Healthy skin biopsies were collected from volunteers residing in a non-endemic area with no history of leishmaniasis at the Department of Dermatology, Ibn Rochd Hospital, in Casablanca, Morocco. Participants were excluded from the study if they had pregnancy, heart disease, diabetes mellitus, or had undergone recent significant operations.

The skin biopsies of sixteen Iranian CL patients with *L. tropica* were taken from the Mashhad Medical School’s CL special clinic, which is located in Mashhad, Iran. All CL patients were in the acute lesion phase for a maximum of 1 year and had received no previous anti-leishmanial medication. Healthy skin biopsies from volunteers residing in a non-endemic area with no history of leishmaniasis were collected from the Noor Eye Hospital in Tehran, Iran. Pregnancy, heart diseases, diabetes mellitus, and recent significant operations were the exclusion criteria from the study.

### 2.3. PCR diagnosis of patient

In Morocco, the swab sampling approach was used to diagnose and identify *Leishmania* species by gently rubbing the skin lesion approximately five times and then storing it at −20°C until DNA extraction using phenol-chloroform extraction followed by ethanol precipitation [13].

Diagnosis of Iranian CL patients was performed using a non-invasive, tape discs sampling method as described previously [14]. DNA samples from the tape discs were extracted using DNAeasy Blood & Tissue kit (QIAGEN, Hilden, Germany).

DNA samples were then subjected to polymerase chain reaction (PCR) amplification of the *Leishmania*-specific internal transcribed spacer ribosomal 1 (ITS1) region using the primer pair L5.8S and LITSR, followed by restriction fragment length polymorphism (RFLP) analysis, as described previously [14,15].

### 2.4. Skin lesion sampling

The skin biopsies from CL patients were taken at the border of the lesion using a 3 mm and 2 mm punch biopsy for CL patients infected with *L. major* (Morocco) and *L. tropica* (Iran), respectively. In addition, both nations provided punch biopsies from healthy skin volunteers. Biopsies were immediately placed in RNA later (Qiagen GmbH, Hilden, Germany) and stored at −20° C until RNA isolation.

### 2.5. RNA isolation

Skin biopsies related to both study groups were homogenized in QIAzol lysis buffer (Qiagen, GmbH, Hilden, Germany) with stainless steel beads and TissueLyser II (Qiagen, GmbH, Hilden, Germany). Total RNAs were isolated using miRNeasy Mini Kit (Qiagen, GmbH, Hilden, Germany), following to the manufacturer’s instructions and stored at −80°C until use. RNA concentrations and purity (A260/A280 ratio 1.8–2.2) were measured using Nanodrop spectrophotometer (Thermo Scientific, USA). RNA integrity (RIN) were evaluated by Agilent 2200 TapeStation (Agilent Technologies, USA).

### 2.6. dcRT-MLPA assay

Quality RNAs were subjected to dcRT-MLPA assay in accordance with the previously described protocol [16]. In brief, a specific reverse transcription (RT) primer that is found directly downstream of the left and right-hand half-probe target sequences was designed for each target-specific sequence. After reverse transcription into cDNA, the left and right half-probes were hybridized to the cDNA at 60 °C overnight. Annealed half-probes were ligated by ligase and amplified by PCR (33 cycles of 30 s/95 °C, 30 s/58 °C, 60 s/72 °C, followed by 1 cycle of 20 min/72 °C). Primers and probes were synthesized by Sigma-Aldrich Chemie (Zwijndrecht, The Netherlands) and MLPA reagents were obtained from MRC-Holland (Amsterdam, The Netherlands). The GeneMapper software tool (Applied Biosystems) was used to examine trace data. Signals below the threshold value for noise cutoff in GeneMapper (in arbitrary units) were assigned the threshold value for noise cut-off. Data were normalized to GAPDH and signals below the threshold value for noise cutoff in GeneMapper (Log2-transformed peak area of 7.64) were assigned the threshold value. The raw and analyzed data were submitted to the Gene Expression Omnibus (GEO) repository (GSE220308). The dcRT-MLPA assay used in the current study included 144 genes associated with innate, adaptive, and inflammatory immune responses (S1 Table).

### 2.7. Data analysis and statistics

The normalized immune-related gene expression data were Log2-transformed before being used in the subsequent statistical analysis. A list of differentially expressed genes (DEGs) between the *L. major*-infected patients, *L. tropica*-infected patients, and their respective healthy volunteer groups was generated using the R (version 4.1.1; http://CRAN.R-project.org/bin/windows/base) package Limma (version 3.48.3) with the empirical Bayes method. The *p*-value of less than 0.05 was considered statistically significant.

Uniform manifold approximation project (UMAP) visualization was created using umap package (version 0.2.10.0), tidyverse package (version 1.3.1.9), magrittr package (version 2.0.1), and ggplot2 package (version 3.3.5). Partial least square discriminant analysis (PLS-DA) plots were generated using mixOmics (version 6.16.3), lattice (version 0.20.44), and ggplot2 (version 3.3.5) packages. The R packages ggplot2 and ggrepel (version 0.9.1) packages were used to generate volcano plots. Heatmap plots were created using gplots (version 3.1.1) and dplyr (version 1.0.7) packages. Putative immune-related panel plots (based on specified panels in S1 Table) were visualized using ggplot2 (version 3.3.5) package.

The Fisher’s exact test was applied to biological sex and lesion number, the One-way ANOVA test was used for age, and Mann-Whitney tests were conducted for lesion size and illness duration in the studied groups. Pearson correlation coefficient test with the GraphPad Prism (GraphPad Software Inc., CA, USA) was applied to identify a correlation between the significant gene expression and clinical data of the studied groups. Correlation *r* values ≥ 0.6 were considered strong correlation and the level of significance was set to *P ≤* 0.05.

## 3. Results

### 3.1. Demographic and clinical profile of Moroccan and Iranian CL patients

Demographic and clinical features of the 14 *L. major*-infected patients and four healthy study participants from Morocco and 16 *L. tropica*-infected patients and 6 healthy study participants from Iran are shown in Table 1. The mean age and lesion size for Moroccan patients were 36.07 years (SEM 6.38) and 5 cm^2^ (SEM 1.76), while for Iranian patients were 43.81 (SEM 3.43) years and 15 cm^2^ (SEM 5.68). However, there are no significant differences in age (across all studied groups), size of lesion, and lesion number between patients caused by the two species. Further, illness duration was 1.48 months (SEM 0.32) for *L. major* and 4.37 months (SEM 0.78) for *L. tropica*-infected patients, indicating significant differences between the studied groups (*P* = 0.002).

**Table 1:** Demographics and clinical profiles of the CL patients and healthy volunteers in Morocco and Iran.

Countries	Morocco		Iran		*P*-value^b^
	CL lesions infected with *L. major* (n = 14)	Healthy volunteers (n = 4)	CL lesions infected with *L. tropica* (n = 16)	Healthy volunteers (n = 6)	
**Biological sex**					
**Female**	8 (57.14%)	–	10 (62.5%)	3 (50%)	0.18
**Male**	6 (42.85%)	4 (100%)	6 (37.5%)	3 (50%)	
**Region**	Ouarzazate Tinghir Zagora	Casablanca	Mashhad	Tehran	–
**Age** ^**a**^	36.07±6.38	27.75±1.18	43.81±3.43	41.18±2.10	0.21
**Lesion size**^**a**^ **(cm**^**2**^)	5 ±1.76	–	15±5.68	–	0.79
**Illness duration**^**a**^ **(month)**	1.48±0.32	–	4.37±0.78	–	0.002^*^
**Lesion number**					
**1**	4 (28.6%)	–	8 (50%)	–	0.23
**≥2**	10 (71.42%)		8 (50%)		

^a^Age, lesion size and illness duration are presented as mean± SEM (standard error of the mean). ^b^*P*-values were calculated using Fisher’s exact test for biological sex and lesion number, One-way ANOVA for age, and Mann-Whitney tests for lesion size and illness duration.

* Difference is statistically significant at *P ≤* 0.05 level.

### 3.2. Overall immunological gene magnitude in Moroccan and Iranian CL patients compared to healthy individuals

**Fig 1:**
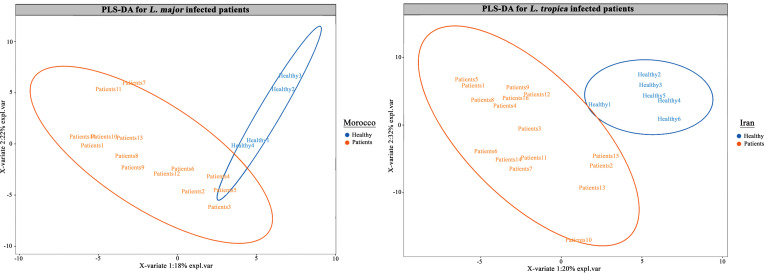
Partial least squares-discriminant analysis (PLS-DA) modeling was applied for the classification of (a) Morocco CL patients infected with *L. major* and (b) Iran CL patients infected with *L. tropica* species compared to healthy volunteers. A supervised global analysis of the 144 immune gene expression data was performed with the multivariate analysis tool PLS-DA. Each data point represents one sample and the circles denote respective group clusters.

Using dcRT-MLPA, the expression of 144 immune response genes was assessed in CL patients from Morocco and Iran who had been infected with *L. major* and *L. tropica*, respectively. UMAP was performed to visualize overall trends in the *L. major* and *L. tropica*-infected patients from Morocco and Iran, respectively, as well as healthy study participants (S1 Fig). UMAP analysis, an unsupervised approach, revealed distinct clustering between *L. tropica*-infected patients and healthy volunteers in the Iranian study group, while the Moroccan *L. major*-infected patients and healthy volunteers showed less clear separation (S1 Fig). To further investigate these group differences, a supervised PLS-DA model was applied to the dcRT-MLPA data due to its ability to present high-dimensional data and identify key variables that drive group separation. The PLS-DA analysis demonstrated clear separation between CL patients and healthy volunteers in both Iranian and Moroccan groups (Fig 1).

**Fig 2:**
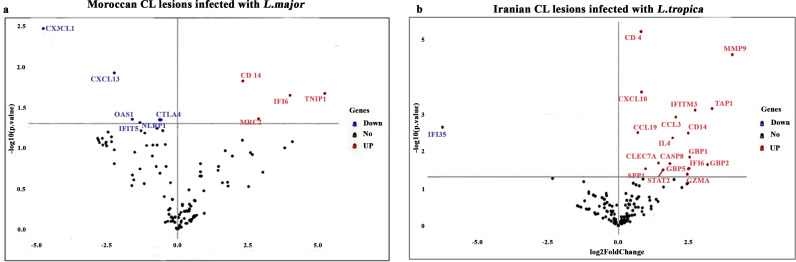
Volcano plot associated with differential immune gene expression in CL patients. Volcano plots displaying significantly regulated genes in CL patients from (a) Morocco due to *L. major* and (b) Iran due to *L. tropica* compared to healthy volunteers. The y-axis corresponds to the *P*-values (-Log10), and the x-axis displays the Log2 fold change values. Using the *P*-value cutoff (*P ≤* 0.05), the over- and under-expressed DEGs are depicted as red and blue dots, respectively. Black dots represent non-significantly different genes.

DEGs based on Limma analysis were identified in the skin lesions of the *L. major* and *L. tropica*-infected patients relative to healthy biopsies’ volunteers. The statistical analysis results for all 144 genes in the Moroccan and Iranian studied groups are presented in the S2 and S3 Tables, respectively. Moreover, a volcano plot in the studied groups (Fig 2a and 2b) showed a total of 10 and 19 differentially expressed genes (*P*-value *≤* 0.05) in the skin lesions of the *L. major* and *L. tropica*-infected patients compared to healthy volunteers, respectively.

**Fig 3:**
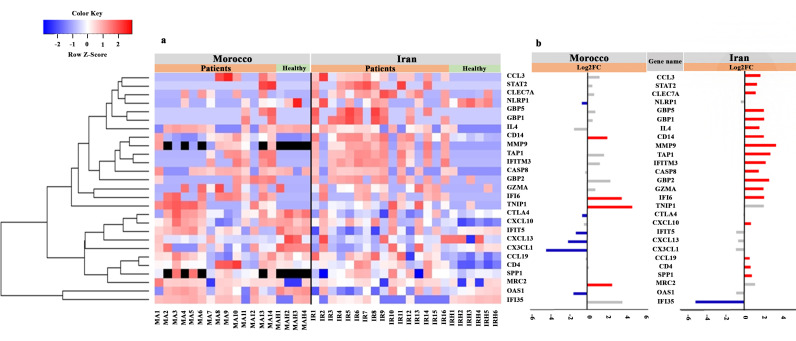
Expression pattern of statistically significantly differentially expressed immune-related genes in CL patients compared to healthy volunteers. (a) A heatmap displaying color-coded expression levels of genes in CL patients from Morocco (MA1-MA14) and Iran (IR1-IR16) infected with *L. major* and *L. tropica*, respectively, as well as healthy samples from each group (MAH1-MAH4; IRH1-IRH6). The heatmap was generated with normalized Log2 transformed dcRT-MLPA data. Only significant genes (10 and 19 DEGs were identified in Morocco and Iran studied groups, respectively) with a *P*-value cutoff (*P ≤* 0.05) were included. It should be noted the CD14 and IFI6 are two common genes between both studied groups. The black color represents no detectable signal in the dcRT-MLPA assay. (b) The bar plot indicates Log2FC of DEGs associated with Morocco and Iran lesions. The red and blue colors of the bar plot represent relative significant increased or decreased expression of the genes in patients compared to healthy samples. The grey box colors indicate no significant changes in the expression of DEGs in the studied groups.

The expression patterns of significantly expressed immune-related genes presented in each CL patient infected with *L. major* and *L. tropica* along with those of healthy volunteers are presented in the heatmap (Fig 3a). In the heatmap, DEGs related to *L. major* and *L. tropica*-infected patients are presented together in order to demonstrate the expression patterns in both studied groups simultaneously. The heatmap illustrates more distinct clustering patterns between the gene expression profiles of *L. tropica*-infected patients than *L. major*-infected patients relative to healthy volunteers. In addition, the bar plot shows that *L. tropica*-infected patients had rather more significant gene expression than *L. major*-infected patients when compared to healthy volunteers (Fig 3b).

### 3.3. Differential expression patterns of significant genes in CL patients infected with *L. major*

Among the 10 DEGs in the *L. major*-infected patients, four genes including CD14, IFI6 (interferon alpha inducible protein 6), TNIP1 (TNF*α*-induced protein 3- (TNFAIP3-) interacting protein 1) and MRC2 (mannose receptor C type 2) were significantly over-expressed compared to healthy volunteer’s group (Figs 2a and 3a). By contrast, the expression of 6 genes including CTLA4 (cytotoxic T-lymphocyte-associated protein 4), CXCL1, CXCL13, IFIT5 (interferon induced protein with tetratricopeptide Repeats 5), OAS1 (2’–5’-oligoadenylate synthetase 1) and NLRP1 (nod-like receptor family pyrin domain containing 1) was significantly decreased in *L. major*-infected patients compared to the healthy volunteer’s group (Figs 2a and 3a).

**Fig 4:**
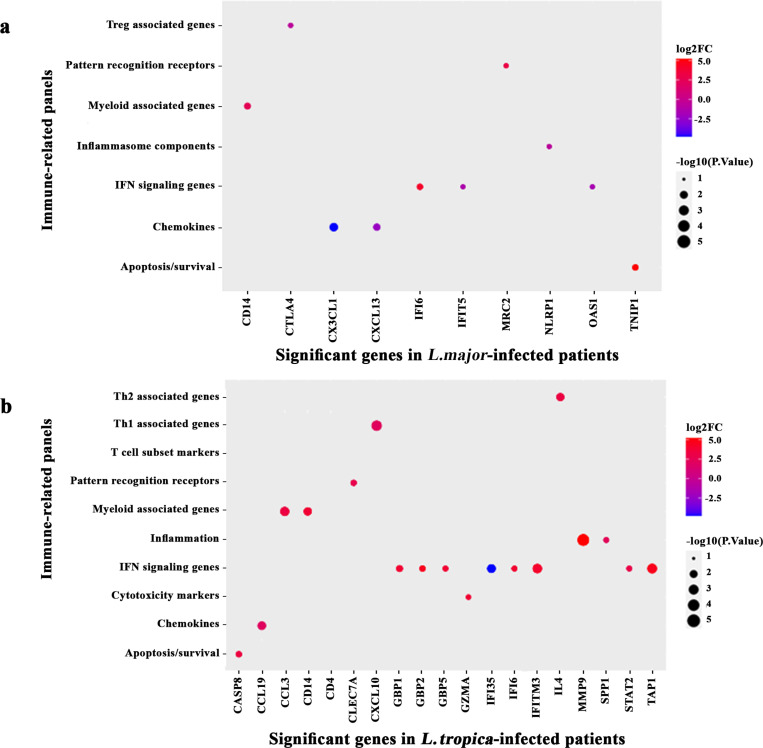
Putative immune-related panels involved in skin lesion of CL patients infected with *L. tropica* and *L. major.* DEGs obtained from (a) Moroccan patients infected with *L. major* and (b) Iranian patients infected with *L. tropica* relative to healthy volunteers. The X-axes indicate significant DEGs (*P ≤* 0.05) and the Y-axes indicate the immune-related panels (specified in S1 Table). The bubbles’ sizes and colors correspond to Log2FC and the *P*-values of significant genes mapped to the indicated panels, respectively. Positive and negative Log2FC denote up- and down-regulated pathways, respectively. Larger bubble sizes refer to more significant genes.

In addition, as depicted in Fig 4a, the DEGs in the lesion of *L. major*-infected patients appeared in seven panels (detailed in S1 Table), including “Chemokines”, “IFN signaling genes”, “Inflammasome components”, “Inflammation”, “Myeloid associated genes”, “Pattern recognition receptors” and “Treg associated genes”.

### 3.4. Elevated gene expression patterns and inflammatory responses in CL patients infected with *L. tropica*

The expression of 18 genes, including CASP8, CD4, CD14, CCL3, CCL19, CXCL10, CLEC7A (C-type lectin domain containing 7A), GBP1 (guanylate-binding protein 1), GBP2, GBP5, GZMA (granzyme A), IFI6, IFITM3 (interferon-induced transmembrane protein 3), IL4, MMP9 (matrix metallopeptidase 9), SPP1 (secreted phosphoprotein 1), STAT2 (signal transducer and activator of transcription 2); and TAP1 (antigen peptide transporter 1) was significantly increased for the *L. tropica*-infected patients, whereas IFI35 expression was reduced relative to those of healthy volunteers (Fig 2b). Our findings revealed that *L. tropica* infection resulted in a higher inflammatory response at the infection site than *L. major* (Fig 3b).

Also, the DEGs of *L. tropica*-infected patients were classified into 10 panels, including “Apoptosis/survival”, “Chemokines”, “Cytotoxicity markers”, “IFN signaling genes”, “Inflammation”, “Myeloid associated genes”, “Pattern recognition receptors”, “T cell subset markers”, “Th1 associated genes” and “Th2 associated genes” (Fig 4b).

Both studied groups showed high enrichment in the number of genes in the IFN signaling panel, as demonstrated in Fig 4a and 4b. Given that IFN signaling plays a crucial role in the host response to *Leishmania* infection in lesions of CL patients. In *L. tropica*-infected patients, the genes including IFI6, TAP1, IFITM3, GBP1, GBP2, and GBP5 were up-regulated. In contrast, expression of OSA1, and IFIT5 in *L. major*-infected patients and IFI35 in *L. tropica*-infected patients was downregulated. The IFI6, as a shared gene between both studied groups, had the same upregulate direction in samples from Morocco and Iran.

### 3.5. Comparative gene expression and clinical correlation in CL patients infected with *L. major* and *L. tropica*

**Fig 5:**
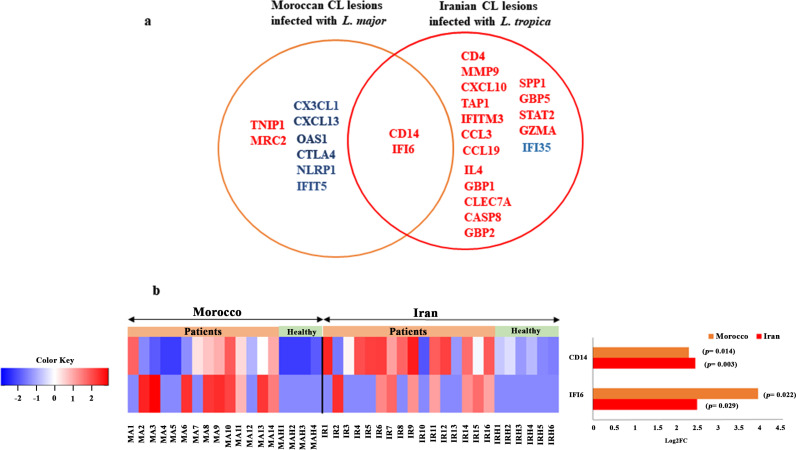
Similarities and differences in gene expression profile in CL patients infected with *L. major* and *L. tropica* species. (a) A Venn diagram represents unique and common DEGs between Moroccan CL lesions infected with *L. major* and Iranian CL lesions infected with *L. tropica* compared to healthy samples. Genes expressed significantly higher or lower than the control are shown in red or blue fonts, respectively. (b) A heatmap displaying color-coded expression levels of genes in CL patients from Morocco (MA1-MA14) and Iran (IR1-IR16) infected with *L. major* and *L. tropica*, respectively, as well as healthy samples from each nation (MAH1-MAH4; IRH1-IRH6). The heatmap was generated with normalized Log2 transformed dcRT-MLPA data. The gene expression pattern of two common genes (CD14 and IFI6) expression in *L. major* and *L. tropica*-infected patients compared to healthy volunteers, as shown by a heatmap on the left and a bar chart on the right.

Among the DEGs of CL patients, 8 and 17 DEGs were uniquely expressed in *L. major* and *L. tropica*-infected patients, respectively (Fig 5a). The genes CD14 and IFI6 were the only two DEGs shared between both studied groups. In Fig 5b, two shared gene expression patterns (heatmap on the left and bar chart on the right) were shown for *L. major*-infected patients and *L. tropica*-infected patients in comparison to healthy volunteers. In CL patients from Morocco and Iran, the corresponding log 2-fold changes for the CD14 and IFI6 genes were 2.31 and 2.47, and 3.98 and 2.51, respectively. The Mann-Whitney U test indicated significant differences in expression levels between *L. major* and *L. tropica*-infected patients for these shared genes (CD14: *P* = 0.014; IFI6: *P* = 0.007).

Further, the Pearson correlation analysis results for the significant gene expression and clinical data of CL patients infected with *L. major* and *L. tropica* are shown in the S4 and S5 Tables, respectively. In the Moroccan samples, the correlation analysis did not yield any statistically significant relationships between gene expression and clinical parameters, specifically illness duration, lesion size, and age. In Iranian CL patients, only GBP5 gene expression exhibited a positive correlation with illness duration (r > 0.65, *P* < 0.005). Other significant genes did not demonstrate a substantial correlation with the clinical data.

## 4. Discussion

CL represents a public health problem worldwide [17]. Gene expression profiling in infectious diseases is an effective approach to determine the immunological pathways that correlate with disease [18]. Genome-wide approaches such as RNA-seq and single-cell RNA-seq are technically challenging, expensive, and complicated in terms of data analysis [16,19]. Herein, we used a multiplex PCR platform (dcRT-MLPA) to investigate the immune responses of selected genes in CL patients from Morocco and Iran. Notably, dcRT-MLPA offers a comparable dynamic range and sensitivity to RNA-seq, but has some limitations, such as probe length and number of pre-selected genes detected per reaction, whereas RNA-seq can potentially analyze the entire transcriptome [20].

Using the dcRT-ML*P*A platform as a robust and user-friendly approach, we evaluated and analyzed the expression of 144 genes implicated in innate, adaptive, and inflammatory panels in skin lesions collected from CL patients infected with *L. major* and *L. tropica*. Our finding highlighted significant distinctions in the clustering patterns of *L. tropica*-infected patients compared to healthy controls, as revealed by both UMAP and PLS-DA analyses. The unsupervised UMAP approach illustrated a clear separation between *L. tropica*-infected patients and healthy volunteers within the Iranian study group, indicating a robust differential expression of genes associated with this infection. In contrast, the Moroccan *L. major*-infected patients exhibited less pronounced clustering, suggesting that the underlying biological responses may differ between these two species.

In the lesion of *L. major*-infected patients, 4 significant genes, including CD14, IFI6, TNIP1 and MRC2 were over-expressed relative to healthy volunteers. CD14 and IFI6 were two common genes with similar expression patterns in both cohorts, and significant differences were also observed between the patient groups. CD14, which is expressed on the surface of myelomonocytic cells or released in a soluble form, mediates a variety of activities in response to infections [21]. As a co-receptor for Toll-like receptor (TLR) 4, CD14 plays a crucial function in innate immunity [22].The expression levels of CD14 on different monocyte subsets may serve as potential biomarkers for CL diagnosis. In cases of visceral leishmaniasis caused by *L. infantum*, elevated CD14 expression has been reported, indicating a strong inflammatory response and suggesting active infection [23,24]. IFI6 is an IFN-stimulated gene (ISG) belongs to the ISG12 gene family, whose expression is controlled by type I IFN [25]. IFI6 is considered as a pro-survival gene because of its high expressed in psoriasis [26], breast cancer [27], colorectal cancer [28], and gastric cancer [29]. IFI6 regulates by Janus tyrosine kinase (JAK)/ STAT signal transduction pathway and through inhibiting the release of cytochrome c from mitochondria delays apoptosis, which plays a protective role during infection [30]. IFI6 has a well-known functional role in the immune system, but its involvement in CL remains enigmatic.

TNIP1 is considered a major repressor of the inflammatory signaling cascade [31]. The expression of the TNIP1 gene was reported in murine macrophages infected with *L. amazonesis*. Further, TNF-related molecules TNIP1 and Tnfaip8l2 were shown to be up- and down-regulated, respectively, indicating regulation of signaling cascades to maintain immunological homeostasis [32]. MRC2 is another induced gene in the *L. major*-infected patients’ group, and it was similarly upregulated in the blood of *L. tropica*-infected patients with active CL disease as shown in our recent report [33].

In addition to the shared IFI6 gene in both lesions’ groups, two additional DEGs (OAS1 and IFIT5) in patients infected with *L. major* and seven additional genes (GBP1, GBP2, GBP5, IFI35, IFITM3, STAT2, and TAP1) in patients infected with *L. tropica* also belong to the IFN signaling pathway.

In the current study, we found that OAS1 and IFIT5 genes in the *L. major*-infected patients were down-regulated compared to control groups. The role of the OAS1 gene has been implicated in the cell cycle-dependent proliferation of epidermal keratinocytes and the induction of type I IFN via control of the Jak/STAT pathway in psoriasis [34]. The role of IFIT5 in human dendritic cells was previously documented in a study in which *L. major* was demonstrated to induce IFIT5 expression, leading to IL-12 production and a robust Th1-type response [35].

Our results presented herein showed that 3 guanylate-binding protein genes, GBP1, GBP2, and GBP5, as well as IFITM3, STAT2, and TAP1 genes were significantly over-expressed in the *L. tropica*-infected patients. It has been shown that dendritic cells derived from the blood of healthy human donors expressed more GBP1 and GBP2 after infection with *L. major* promastigotes, whereas dendritic cells infected with *L. donovani* expressed more GBP1 [36]. GBP5 mRNA expression was shown to be up-regulated in skin lesions of both *L. braziliensis* [37] and *L. tropica*- infected patients [38]. Importantly, anti-*Leishmania* responses mediated by GBPs have been identified in mouse and human non-phagocytic cells infected with *L. donovani* via mechanisms distinct from those observed in macrophages [39]. The complexities of the GBPs involvement in CL disease still warrant further investigation. Our current study found a positive correlation between the GBP5 gene and illness duration in the lesion of Iranian CL infected with *L. tropica*, which suggests that probably GBP5 have an important role in controlling *L. tropica* in the lesion of patients [17,40]. Haldar *et al.* [39] highlights the crucial role of GBPs in the immune response against *L. donovani*. The study demonstrates that GBPs enhance the ability of nonphagocytic cells to inhibit parasite growth through mechanisms that do not rely on traditional parasitophorous vacuole targeting. This suggests that GBPs could be valuable targets for developing innovative treatment strategies for leishmaniasis. However, further experiments are needed to determine whether GBP5 or other GBPs serve as effectors of IFNγ, or if they operate independently of other cytokine networks [17].

It is worth noting that both CL diseases caused by parasites *L. major* and *L. tropica* are transmitted through the bite of sand flies, but their characteristics, such as the duration of infection, might vary in different regions [41]. Significant differences in illness duration were observed between the Moroccan and Iranian patients of our studied groups, possibly indicating that the duration of the disease is affected by the parasite species causing the infection.

Using whole genome RNA-seq, we have recently reported expression of the IFN signaling pathway among the top five clusters of enriched molecular pathways in the ulcerative and non-ulcerative skin lesions of patients infected with *L*. *tropica* [11]. Moreover, Amorim *et al*. recently reported on RNA-seq transcriptomics analysis of blood from *L*. *braziliensis*-infected patients showing a significant ISG signature related to monocyte and macrophage, suggesting the involvement of IFN signaling and monocytes in CL [42]. Further, using dcRT-MLPA we recently reported significant alterations in the gene expression of the IFN-signaling pathway in the whole blood of CL patients infected with *L. tropica* as well as asymptomatic and healed individuals [33]. Collectively, these findings point to the involvement of type I IFN signaling in both skin lesion and blood during CL.

We could also herein report high expression levels of CASP8, GZMA, and MMP9 genes in the *L. tropica* skin lesions compared to healthy volunteers. In line with this finding, the CASP8 protein with an important role in macrophage apoptosis [43], was shown in our recent RNA-seq study to be uniquely expressed in the ulcerative CL lesion group [11]. Similar to the results presented herein, microarray transcriptional analysis of skin lesions of *L. braziliensis*-infected patients revealed that GZMA ranks in the top 10-induced genes [37]. Further, increased expression of GZMA in the lesions of *L. braziliensis*-infected patients with late-stage CL versus early-stage CL suggests that CD8^+^ T-cell recruitment and GZMA expression of those cells is implicated in human CL lesion progression [44]. In a separate study conducted by Rodrigues *et al*., the increased expression of GZMA, along with other genes like GZMB, PRF1, and CD8B, in Moroccan lesions resulting from both *L. major* and *L. tropica*, suggest an important role in CD8 T cell immune responses [12]. MMP9 has also been noted as one of the most up-regulated genes in our transcriptome analysis of *L. tropica* CL lesions [38]. A previous study in the CL skin lesions due to *L. braziliensis* infection showed that TNF could enhance the tissue damage by an increase in gene expression of MMP9 [45].

Significant over-expression of chemokines CCL3, CCL19, and CXCL10, which are involved in inflammation and the attraction of immune effector cells, were noted in the skin lesions of *L. tropica*-infected patients. Further, CLEC7A, a pattern recognition receptor, expressed by myeloid phagocytes was found to be over-expressed in the lesion of CL *L. tropica* patients. This inflammatory milieu may contribute to the migration of myeloid phagocyte cells to the skin, which in turn may facilitate parasite phagocytosis and inflammation-induced skin disease [46]. Consistent with our study, the CCL3 gene, which functions as an inflammatory chemokine, exhibited a similar expression pattern in Moroccan lesions infected with *L. major* and *L. tropica* [12]. Furthermore, Antonia *et al*. demonstrated in various human cell lines that *L. major* infection reduces CXCL10 protein levels while elevating CXCL10 mRNA levels [47]. The suppression occurs due to the proteolytic activity of the virulence factor known as glycoprotein-63 (GP63). This finding suggests that the suppression of CXCL10 is a shared mechanism of immune evasion [47]. Additionally, it has also been demonstrated that chemokines and their receptors play a role in parasite control by promoting Th1 responses and the outcome of disease [46].

Further, our results showed a relative under-expression of chemokine genes CX3CL1, CXCL13, CTLA4, and NLRP1 in the lesion of *L. major*-infected patients. Nevertheless, over-expression of CXCL13 involved in recruitment of inflammatory immune cells [48] and NLRP1 gene as inflammasome-associated markers [49], has been reported in skin lesions of CL patients caused by *L. braziliensis* as well as CTLA4 gene expression in *L. infantum*-infected mice [50]. CTLA4 plays a pivotal role as a regulatory checkpoint in the immune response against *Leishmania* infections, and its inhibition has been identified as a promising therapeutic strategy. Blocking CTLA4 leads to enhanced activation of T cells and increased production of cytokines, which together strengthen the host’s defense mechanisms against the parasite. Evidence suggests that treatment with anti-CTLA4 monoclonal antibodies can lead to a significant decrease in parasite load by encouraging Th1 immune responses [51]. While it is not possible to directly compare gene expression across RNA-Seq and dcRT-MLPA datasets, we performed an overlap of the DEGs in CL patients investigated herein to those of the DEGs determined by RNA-seq, as previously published for Iranian patients infected with *L. tropica* [11]. We found 6 common genes (CXCL10, MMP9, GBP1, GBP2, GBP5, and TAP1) in the lesion of *L. tropica* infected patients, but only one common gene (MRC2) in the lesion of *L. major* infected patients. This suggests the parasite species determine the immune response at the lesion site.

It is worth noting that a significant aspect of our study is the direct comparison of the transcriptional profiles of CL lesions from two different countries (Morocco and Iran) caused by infections with two separate *Leishmania* species. Rodrigues *et al*., reported the same comparison of the transcriptome in patients’ lesions caused by both *L. major* and *L. tropica*, but only in Moroccan patients [12]. Our results suggest that the transcriptional profiles of CL caused by *L. major* and *L. tropica* are distinct. However, these findings, in contrast to Rodrigues *et al*. publication data [12], suggest that both parasite species and ancestry may influence the interpretation of differential gene expression studies.

In summary, we identified 144 inflammatory-related genes by dcRT-MLPA method in both the Moroccan and Iranian patient populations. Patients with lesions induced by *L. major* and *L. tropica* shared two upregulated DEGs, CD14 and IFI6, and the immune-related panel showed that both lesion groups had significant alterations in the expression of genes involved in the IFN signaling pathway. Further, the higher number of significantly expressed genes and related panels in CL patients infected with *L. tropica* suggests that *L. tropica* infection resulted in a stronger inflammatory response at the infection site than *L. major*. Although, larger sample sizes are required to provide stronger and more reliable evidence. The clinical management and distinctions in species-specific gene expression offer valuable insights for comprehending the disease. These findings present new insight into the comparative immune signatures of *L. major* and *L. tropica* in the lesions of CL patients, which are pivotal for future research endeavors aiming to establish effective diagnostic or therapeutic strategies for CL disease.

## Supporting information

S1 Fig Uniform manifold approximation project (UMAP) of immune-related gene expression in the skin lesions of CL patients and healthy controls biopsies. The UMAP plot (as an unsupervised model) displays the separation between the subjects included in the study, each data point represents one sample. Data points have been color-coded according to (a) Morocco CL lesions infected with *L. major* and (b) Iran CL lesions infected with *L. tropica* (blue circles) compare to healthy (red circles).
(TIF)

S1 Table List of genes assayed by dcRT-MLPA. (PDF)

S2 Table The Limma package was applied to evaluate the differentially expressed genes (DEGs) for Morocco CL patients relative to healthy volunteers. Red highlighted genes represent significant DEGs.
(PDF)

S3 Table The Limma package was applied to evaluate the DEGs for Iran CL patients relative to healthy volunteers. Red highlighted genes represent significant DEGs.
(PDF)

S4 Table Pearson correlation (*r*) between the significant gene expression and clinical data for Morocco CL patients, and associated *P*-values. (PDF)

S5 Table Pearson correlation (*r*) between the significant gene expression and clinical data for Iran CL patients, and associated *P*-values. (PDF)

## References

[1] Knight CA, Harris DR, Alshammari SO, Gugssa A, Young T, Lee CM. Leishmaniasis: recent epidemiological studies in the Middle East. Front Microbiol. 2023;13:1052478. doi: 10.3389/fmicb.2022.1052478 36817103 PMC9932337

[2] Ghatee MA, Taylor WR, Karamian M. The geographical distribution of cutaneous leishmaniasis causative agents in Iran and its neighboring countries, a review. Front Public Health. 2020;8:11. doi: 10.3389/fpubh.2020.00011 32133334 PMC7039857

[3] Abadías-Granado I, Diago A, Cerro P, Palma-Ruiz A, Gilaberte Y. Cutaneous and mucocutaneous leishmaniasis. Actas Dermo-Sifiliográficas (English Edition). 2021;112(7):601–18. doi: INSERT_DOI_HERE 10.1016/j.adengl.2021.05.011 34045157

[4] Tabbabi A. Review of leishmaniasis in the Middle East and North Africa. Afr Health Sci. 2019;19(1):1329–37. doi: 10.4314/ahs.v19i1.4 31148958 PMC6531937

[5] Pigott DM, Bhatt S, Golding N, Duda KA, Battle KE, Brady OJ, et al. Global distribution maps of the leishmaniases. Elife. 2014;3:e02851. doi: 10.7554/eLife.02851 24972829 PMC4103681

[6] Masoudzadeh N, Mizbani A, Rafati S. Transcriptomic profiling in cutaneous leishmaniasis patients. Expert Rev Proteomics. 2020;17(7–8):533–41. doi: 10.1080/14789450.2020.1812390 32886890

[7] Cantacessi C, Dantas-Torres F, Nolan MJ, Otranto D. The past, present, and future of *Leishmania* genomics and transcriptomics. Trends Parasitol. 2015;31(3):100–8. doi: 10.1016/j.pt.2014.12.012 25638444 PMC4356521

[8] Mears ER, Modabber F, Don R, Johnson GE. A review: the current in vivo models for the discovery and utility of new anti-leishmanial drugs targeting cutaneous leishmaniasis. PLoS Negl Trop Dis. 2015;9(9):e0003889. doi: 10.1371/journal.pntd.0003889 26334763 PMC4559374

[9] Singhania A, Wilkinson RJ, Rodrigue M, Haldar P, O’Garra A. The value of transcriptomics in advancing knowledge of the immune response and diagnosis in tuberculosis. Nat Immunol. 2018;19(11):1159–68. doi: 10.1038/s41590-018-0225-9 30333612 PMC6554194

[10] Montoya DJ, Andrade P, Silva BJA, Teles RMB, Ma F, Bryson B, et al. Dual RNA-Seq of human leprosy lesions identifies bacterial determinants linked to host immune response. Cell Rep. 2019;26(13):3574–3585.e3. doi: 10.1016/j.celrep.2019.02.109 30917313 PMC6508871

[11] Masoudzadeh N, Östensson M, Persson J, Mashayekhi Goyonlo V, Agbajogu C, Taslimi Y, et al. Molecular signatures of anthroponotic cutaneous leishmaniasis in the lesions of patients infected with *Leishmania tropica*. Sci Rep. 2020;10(1):16198. doi: 10.1038/s41598-020-72671-7 33004861 PMC7529897

[12] Rodrigues V, André S, Maksouri H, Mouttaki T, Chiheb S, Riyad M, et al. Transcriptional analysis of human skin lesions identifies tryptophan-2,3-deoxygenase as a restriction factor for cutaneous *Leishmania*. Front Cell Infect Microbiol. 2019;9:338. doi: 10.3389/fcimb.2019.00338 31637219 PMC6788307

[13] Daoui O, Ait Kbaich M, Mhaidi I, El Kacem S, Hjiyej Andaloussi L, Akarid K, et al. The role of sampling by cotton swab in the molecular diagnosis of cutaneous leishmaniasis. Transbound Emerg Dis. 2021;68(4):2287–94. doi: 10.1111/tbed.13886 33094519

[14] Taslimi Y, Sadeghipour P, Habibzadeh S, Mashayekhi V, Mortazavi H, Müller I, et al. A novel non-invasive diagnostic sampling technique for cutaneous leishmaniasis. PLoS Negl Trop Dis. 2017;11(7):e0005750. doi: 10.1371/journal.pntd.0005750 28704463 PMC5526608

[15] Schönian G, Nasereddin A, Dinse N, Schweynoch C, Schallig HDFH, Presber W, et al. PCR diagnosis and characterization of *Leishmania* in local and imported clinical samples. Diagn Microbiol Infect Dis. 2003;47(1):349–58. doi: 10.1016/s0732-8893(03)00093-2 12967749

[16] Haks MC, Goeman JJ, Magis-Escurra C, Ottenhoff THM. Focused human gene expression profiling using dual-color reverse transcriptase multiplex ligation-dependent probe amplification. Vaccine. 2015;33(40):5282–8. doi: 10.1016/j.vaccine.2015.04.054 25917681

[17] Lipoldová M, Sohrabi Y. Role of interferon-induced GTPases in leishmaniasis. PLoS Negl Trop Dis. 2022;16(1):e0010093. doi: 10.1371/journal.pntd.0010093 35085246 PMC8794175

[18] Amorim CF, Novais FO, Nguyen BT, Misic AM, Carvalho LP, Carvalho EM, et al. Variable gene expression and parasite load predict treatment outcome in cutaneous leishmaniasis. Sci Transl Med. 2019;11(519):eaax4204. doi: 10.1126/scitranslmed.aax4204 31748229 PMC7068779

[19] Jovic D, Liang X, Zeng H, Lin L, Xu F, Luo Y. Single-cell RNA sequencing technologies and applications: a brief overview. Clin Transl Med. 2022;12(3):e694. doi: 10.1002/ctm2.694 35352511 PMC8964935

[20] Sivakumaran D, Jenum S, Vaz M, Selvam S, Ottenhoff THM, Haks MC, et al. Combining host-derived biomarkers with patient characteristics improves signature performance in predicting tuberculosis treatment outcomes. Commun Biol. 2020;3(1):359. doi: 10.1038/s42003-020-1087-x 32647325 PMC7347567

[21] Zanoni I, Granucci F. Role of CD14 in host protection against infections and in metabolism regulation. Front Cell Infect Microbiol. 2013;3:32. doi: 10.3389/fcimb.2013.00032 23898465 PMC3721004

[22] Costa GC, Rocha MO da C, Souza PEA de, Melo DFS, Moreira PR, Gollob KJ, et al. CD14 genotype and functional dichotomy of CD14+ and CD14- cells are associated with activated immune response and development of Chagas dilated cardiomyopathy. Mem Inst Oswaldo Cruz. 2020;115:e200110. doi: 10.1590/0074-02760200110 33146244 PMC7592494

[23] Viana AG, Magalhães LMD, Giunchetti RC, Dutra WO, Gollob KJ. Infection of human monocytes with *Leishmania infantum* strains induces a downmodulated response when compared with infection with *Leishmania braziliensis*. Front Immunol. 2018;8:1896. doi: 10.3389/fimmu.2017.01896 29358935 PMC5766652

[24] Picard M, Soundaramourty C, Silvestre R, Estaquier J, André S. *Leishmania infantum* infection of primary human myeloid cells. Microorganisms. 2022;10(6):1243. doi: 10.3390/microorganisms10061243 35744760 PMC9230042

[25] Sajid M, Ullah H, Yan K, He M, Feng J, Shereen MA, et al. The functional and antiviral activity of interferon alpha-inducible IFI6 against hepatitis B virus replication and gene expression. Front Immunol. 2021;12:634937. doi: 10.3389/fimmu.2021.634937 33868257 PMC8047077

[26] Szegedi K, Sonkoly E, Nagy N, Németh IB, Bata-Csörgo Z, Kemény L, et al. The anti-apoptotic protein G1P3 is overexpressed in psoriasis and regulated by the non-coding RNA, PRINS. Exp Dermatol. 2010;19(3):269–78. doi: 10.1111/j.1600-0625.2010.01066.x 20377629

[27] Cheriyath V, Kuhns MA, Jacobs BS, Evangelista P, Elson P, Downs-Kelly E, et al. G1P3, an interferon- and estrogen-induced survival protein contributes to hyperplasia, tamoxifen resistance and poor outcomes in breast cancer. Oncogene. 2012;31(17):2222–36. doi: 10.1038/onc.2011.393 21996729

[28] Lee J, Li L, Gretz N, Gebert J, Dihlmann S. Absent in Melanoma 2 (AIM2) is an important mediator of interferon-dependent and -independent HLA-DRA and HLA-DRB gene expression in colorectal cancers. Oncogene. 2012;31(10):1242–53. doi: 10.1038/onc.2011.320 21804607 PMC3307062

[29] Cheriyath V, Glaser KB, Waring JF, Baz R, Hussein MA, Borden EC. G1P3, an IFN-induced survival factor, antagonizes TRAIL-induced apoptosis in human myeloma cells. J Clin Invest. 2007;117(10):3107–17. doi: 10.1172/JCI31122 17823654 PMC1964509

[30] Qi Y, Li Y, Zhang Y, Zhang L, Wang Z, Zhang X, et al. IFI6 inhibits apoptosis via mitochondrial-dependent pathway in dengue virus 2 infected vascular endothelial cells. PLoS One. 2015;10(8):e0132743. doi: 10.1371/journal.pone.0132743 26244642 PMC4526556

[31] Shamilov R, Aneskievich BJ. TNIP1 in autoimmune diseases: regulation of toll-like receptor signaling. J Immunol Res. 2018;2018:3491269. doi: 10.1155/2018/3491269 30402506 PMC6192141

[32] Aoki JI, Muxel SM, Zampieri RA, Müller KE, Nerland AH, Floeter-Winter LM. Differential immune response modulation in early *Leishmania amazonensis* infection of BALB/c and C57BL/6 macrophages based on transcriptome profiles. Sci Rep. 2019;9(1):19841. doi: 10.1038/s41598-019-56305-1 31882833 PMC6934472

[33] Bahrami F, Masoudzadeh N, Van Veen S, Persson J, Lari A, Sarvnaz H, et al. Blood transcriptional profiles distinguish different clinical stages of cutaneous leishmaniasis in humans. Mol Immunol. 2022;149:165–73. doi: 10.1016/j.molimm.2022.07.008 35905592

[34] Huang Y-Z, Zheng Y-X, Zhou Y, Xu F, Cui Y-Z, Chen X-Y, et al. OAS1, OAS2, and OAS3 contribute to epidermal keratinocyte proliferation by regulating cell cycle and augmenting IFN-1‒induced jak1‒signal transducer and activator of transcription 1 phosphorylation in psoriasis. J Invest Dermatol. 2022;142(10):2635–2645.e9. doi: 10.1016/j.jid.2022.02.018 35305973

[35] Favila MA, Geraci NS, Zeng E, Harker B, Condon D, Cotton RN, et al. Human dendritic cells exhibit a pronounced type I IFN signature following *Leishmania* major infection that is required for IL-12 induction. J Immunol. 2014;192(12):5863–72. doi: 10.4049/jimmunol.1203230 24808365 PMC4052223

[36] Jayakumar A, Donovan MJ, Tripathi V, Ramalho-Ortigao M, McDowell MA. Leishmania major infection activates NF-kappaB and interferon regulatory factors 1 and 8 in human dendritic cells. Infect Immun. 2008;76(5):2138–48. doi: 10.1128/IAI.01252-07 18316378 PMC2346706

[37] Novais FO, Carvalho LP, Passos S, Roos DS, Carvalho EM, Scott P, et al. Genomic profiling of human *Leishmania braziliensis* lesions identifies transcriptional modules associated with cutaneous immunopathology. J Invest Dermatol. 2015;135(1):94–101. doi: 10.1038/jid.2014.305 25036052 PMC4268311

[38] Masoudzadeh N, Mizbani A, Taslimi Y, Mashayekhi V, Mortazavi H, Sadeghipour P, et al. *Leishmania tropica* infected human lesions: whole genome transcription profiling. Acta Trop. 2017;176 236–41. doi: 10.1016/j.actatropica.2017.08.016 28842129

[39] Haldar AK, Nigam U, Yamamoto M, Coers J, Goyal N. Guanylate binding proteins restrict *Leishmania donovani* growth in nonphagocytic cells independent of parasitophorous vacuolar targeting. mBio. 2020;11(4):e01464-20. doi: 10.1128/mBio.01464-20 32723921 PMC7387799

[40] Tretina K, Park E-S, Maminska A, MacMicking JD. Interferon-induced guanylate-binding proteins: guardians of host defense in health and disease. J Exp Med. 2019;216(3):482–500. doi: 10.1084/jem.20182031 30755454 PMC6400534

[41] Rostamian M, Niknam HM. *Leishmania tropica*: what we know from its experimental models. Adv Parasitol. 2019;104:1–38. doi: 10.1016/bs.apar.2018.11.001 31030767

[42] Amorim C, Novais F, Nguyen B, Nascimento M, Lago J, Lago A. Localized skin inflammation during cutaneous leishmaniasis drives a chronic, systemic IFN-γ signature. medRxiv. 2020. doi: 10.1101/2020.12.03.20240978 PMC8043375 33793565

[43] DaMata JP, Mendes BP, Maciel-Lima K, Menezes CAS, Dutra WO, Sousa LP, et al. Distinct macrophage fates after in vitro infection with different species of *Leishmania*: induction of apoptosis by *Leishmania* (*Leishmania*) *amazonensis*, but Not by *Leishmania* (Viannia) *guyanensis*. PLoS One. 2015;10(10):e0141196. doi: 10.1371/journal.pone.0141196 26513474 PMC4626090

[44] Faria DR, Souza PEA, Durães FV, Carvalho EM, Gollob KJ, Machado PR, et al. Recruitment of CD8(+) T cells expressing granzyme A is associated with lesion progression in human cutaneous leishmaniasis. Parasite Immunol. 2009;31(8):432–9. doi: 10.1111/j.1365-3024.2009.01125.x 19646207 PMC2764276

[45] Campos TM, Passos ST, Novais FO, Beiting DP, Costa RS, Queiroz A, et al. Matrix metalloproteinase 9 production by monocytes is enhanced by TNF and participates in the pathology of human cutaneous leishmaniasis. PLoS Negl Trop Dis. 2014;8(11):e3282. doi: 10.1371/journal.pntd.0003282 25393535 PMC4230914

[46] Oghumu S, Lezama-Dávila CM, Isaac-Márquez AP, Satoskar AR. Role of chemokines in regulation of immunity against leishmaniasis. Exp Parasitol. 2010;126(3):389–96. doi: 10.1016/j.exppara.2010.02.010 20206625 PMC3661772

[47] Antonia AL, Gibbs KD, Trahair ED, Pittman KJ, Martin AT, Schott BH, et al. Pathogen evasion of chemokine response through suppression of CXCL10. Front Cell Infect Microbiol. 2019;9:280. doi: 10.3389/fcimb.2019.00280 31440475 PMC6693555

[48] Fantecelle CH, Covre LP, Garcia de Moura R, Guedes HL de M, Amorim CF, Scott P, et al. Transcriptomic landscape of skin lesions in cutaneous leishmaniasis reveals a strong CD8+ T cell immunosenescence signature linked to immunopathology. Immunology. 2021;164(4):754–65. doi: 10.1111/imm.13410 34432883 PMC8561102

[49] Gupta G, Santana AKM, Gomes CM, Turatti A, Milanezi CM, Bueno Filho R, et al. Inflammasome gene expression is associated with immunopathology in human localized cutaneous leishmaniasis. Cell Immunol. 2019;341:103920. doi: 10.1016/j.cellimm.2019.04.008 31078283

[50] Ontoria E, Hernández-Santana YE, González-García AC, López MC, Valladares B, Carmelo E. Transcriptional profiling of immune-related genes in *Leishmania infantum*-infected mice: identification of potential biomarkers of infection and progression of disease. Front Cell Infect Microbiol. 2018;8:197. doi: 10.3389/fcimb.2018.00197 30013952 PMC6036295

[51] Murphy ML, Cotterell SE, Gorak PM, Engwerda CR, Kaye PM. Blockade of CTLA-4 enhances host resistance to the intracellular pathogen, *Leishmania donovani*. J Immunol. 1998;161(8):4153–60. doi: 10.4049/jimmunol.161.8.4153 9780188

