# Supplementary figures and images for: Comparative gene expression pattern of immune-related genes using dual-color RT-MLPA in the lesions of cutaneous leishmaniasis caused by L. major and L. tropica
Source: PLoS Negl Trop Dis. 2025 Mar 18;19(3):e0012812. doi: 10.1371/journal.pntd.0012812 (PMC11918365; doi:10.1371/journal.pntd.0012812)

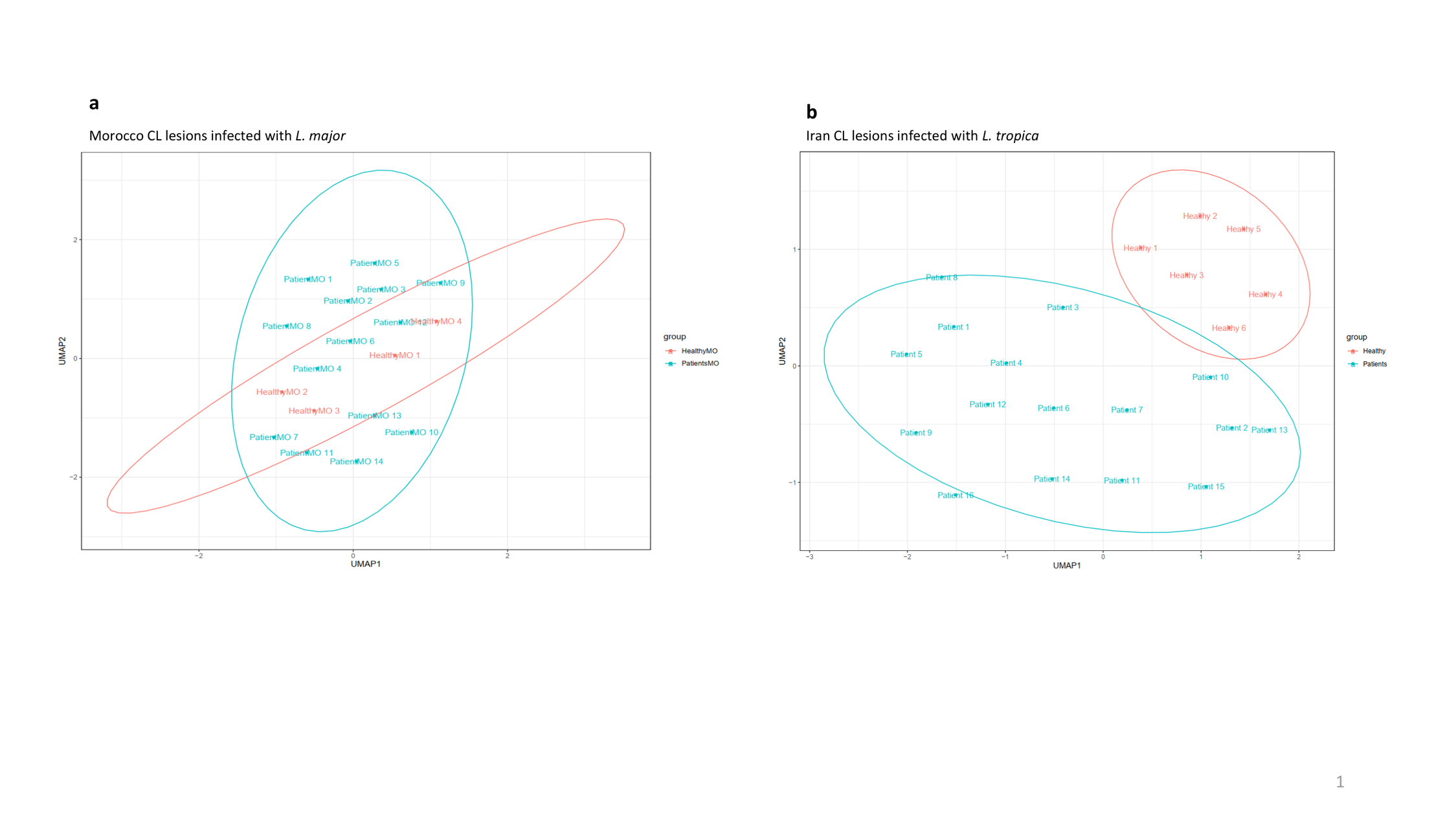

Supplement: S1 Fig — The UMAP plot (as an unsupervised model) displays the separation between the subjects included in the study, each data point represents one sample. Data points have been color-coded according to (a) Morocco CL lesions infected with L. major and (b) Iran CL lesions infected with L. tropica (blue circles) compare to healthy (red circles). (TIF) [file pntd.0012812.s001.tif]
