# Supplementary material for: Comparative gene expression pattern of immune-related genes using dual-color RT-MLPA in the lesions of cutaneous leishmaniasis caused by L. major and L. tropica
Source: PLoS Negl Trop Dis. 2025 Mar 18;19(3):e0012812. doi: 10.1371/journal.pntd.0012812 (PMC11918365; doi:10.1371/journal.pntd.0012812)
Supplement: S1 Table — (PDF) [file pntd.0012812.s002.pdf]

S1 Table. List of genes assayed by dcRT-MLPA.

Innate, adaptive and inflammatory immune responses	Gene Symbol
**Immune cell subset markers**	*CD19;NCAM1*
**T cell subset markers**	*AIRE;CCR7;CD3E;CD4;CD8A;IL7R;PTPRCv1 PTPRCv2*
**Th1 associated genes**	*CXCL10;IFNG;IL1B;IL2;IL15;TBX21;TNF*
**Th2 associated genes**	*GATA3;IL4;IL4δ2;IL5;IL6;IL10;IL13*
**Th9 associated genes**	*IL9*
**Th17 associated genes**	*IL17A;IL22RA1;RORC*
**T_{reg} associated genes**	*CCL4;CTLA4;FOXP3;IL2RA;LAG3;TGFB1; TNFRSF18*
**Cytotoxicity markers**	*GNLY;GZMA;GZMB;PRF1*
**Apoptosis/Survival**	*CASP8;BCL2;FASLG;FLCN1;TNFRSF1A; TNFRSF1B*
**Myeloid associated genes**	*CCL2;CCL3;CCL5;CCL22;CD14;CD163;CXCL13; IL12A;IL12B;IL23A;FPR1*
**Cell activation**	*HCK;LYN;SLAMF7*
**IFN signaling genes**	*CD274;FCGR1A;GBP1;GBP2;GBP5;IFI6;IFI16; IFI35;IFI44;IFI44L;IFIH1;IFIT2;IFIT3;IFIT5; IFITM1/3;INDO;IRF7;OAS1;OAS2;OAS3;SOCS1; STAT1;STAT2;TAP1;TAP2*
**Inflammation**	*DSE;MMP9;SPP1;TIMP2;TNIP1*
**Intracellular transport**	*KIF1B;SEC14L1*
**Chemokines**	*CCL11;CCL13;CCL19;CXCL9;CX3CL1*
**Pattern recognition receptors**	*CD209;CLEC7A;MRC1;MRC2;NOD1;NOD2;TLR1; TLR2;TLR3;TLR4;TLR5;TLR6;TLR7;TLR8;TLR9; TLR10*
**Inflammasome components**	*NLRC4;NLRP1;NLRP2;NLRP3;NLRP4;NLRP6; NLRP7;NLRP10;NLRP11;NLRP12;NLRP13*
**Cell Growth/Proliferation**	*AREG;BMP6;EGF;TGFBR2;VEGF*
**Small GTPases/(Rho) GTPase activating proteins**	*ASAP1;RAB13;RAB24;RAB33A;TAGAP;TBC1D7*
**Anti-microbial activity**	*BPI;LTF*
**Mitochondrial stress/Proteasome**	*HPRT*
**E3 ubiquitin protein ligases**	*NEDD4L*
**Scavenger receptors**	*MARCO*
**Transcriptional regulators/Activators**	*CAMTA1;TWIST1;ZNF331;ZNF532*
**G protein-coupled receptors**	*BLR1*
**Reference genes**	*GAPDH*
