# Supplementary material for: Comparative gene expression pattern of immune-related genes using dual-color RT-MLPA in the lesions of cutaneous leishmaniasis caused by L. major and L. tropica
Source: PLoS Negl Trop Dis. 2025 Mar 18;19(3):e0012812. doi: 10.1371/journal.pntd.0012812 (PMC11918365; doi:10.1371/journal.pntd.0012812)
Supplement: S2 Table — Red highlighted genes represent significant DEGs. (PDF) [file pntd.0012812.s003.pdf]

S2 Table. The Limma package was applied to evaluate the differentially-expressed genes (DEGs) for Morocco CL patients relative to controls. Red highlighted genes represents significant DEGs.

Gene name	Log2FC	Average expression	*p*. value	Adj. *p*. value
AIRE	0.0525	13.08833	0.975983	1
AREG	-0.16571	9.646111	0.790908	1
ASAP1	-2.25286	14.07278	0.105859	0.457149
BCL2	-0.76679	7.966111	0.168833	0.557892
BLR1	-0.7325	12.70778	0.057248	0.440385
BMP6	-2.83893	16.76944	0.075998	0.440385
BPI	0	12.242	1	1
CAMTA1	-1.62464	8.923889	0.292833	0.717068
CASP8	-0.24071	10.22778	0.803849	1
CCL11	-2.64464	16.54556	0.092437	0.440385
CCL13	1.78E-15	10.141	1	1
CCL19	-0.18179	13.19111	0.434273	0.964196
CCL2	0.04	7.671111	0.727646	1
CCL22	0.477857	13.88667	0.52544	1
CCL3	1.430714	8.752778	0.187967	0.588417
CCL4	3.55E-15	12.472	1	1
CCL5	-1.18714	10.80167	0.275858	0.696905
CCR7	0.589643	17.65611	0.712974	1
CD14	2.316786	9.544444	0.014958	0.440385
CD163	-1.78E-15	13.544	1	1
CD19	-3.55E-15	9.877	1	1
CD209	0.408214	8.245	0.327967	0.774217
CD274	-0.44679	9.415	0.823361	1
CD3E	0	10.251	1	1
CD4	0.1675	12.86778	0.51244	1
CD8A	0.563571	14.87333	0.706819	1
CLEC7A	0.786429	8.331667	0.223589	0.61917
CTLA4	-0.59429	14.61778	0.045066	0.440385
CX3CL1	-4.76893	11.76833	0.00339	0.440385
CXCL10	-0.40071	13.96333	0.169531	0.557892
CXCL13	-2.24036	12.76	0.011959	0.440385
CXCL9	-2.67393	18.66278	0.075838	0.440385
DSE	-8.88E-16	7.64	1	1
EGF	-2.77464	16.09944	0.085931	0.440385
FASLG	1.78E-15	11.215	1	1
FCGR1A	-1.78E-15	13.244	1	1
FLCN1	-0.16929	10.53333	0.736392	1
FOXP3	-0.00786	12.41389	0.996095	1
FPR1	-1.55143	10.29833	0.247679	0.672938
GATA3	0.583214	14.90111	0.680907	1
GBP1	0.612143	8.116111	0.442559	0.964196
GBP2	2.659286	9.708333	0.120722	0.467518
GBP5	0.927143	8.361111	0.338225	0.785555

**GNLY**	0.670357	14.31889	0.676881	1
**GZMA**	0.946786	10.39889	0.641245	1
**GZMB**	0.611429	18.37556	0.702615	1
**HCK**	-1.59821	13.56444	0.215402	0.61917
**HPRT**	0.342857	7.906667	0.600604	1
**IFI16**	-1.10393	15.96889	0.093129	0.440385
**IFI35**	4.07	16.68556	0.083508	0.440385
**IFI44**	2.289286	9.420556	0.080947	0.440385
**IFI44L**	-1.295	13.39778	0.0616	0.440385
**IFI6**	3.981429	10.73667	0.022646	0.440385
**IFIH1**	-2.34536	15.51333	0.073409	0.440385
**IFIT2**	0.618571	8.121111	0.448619	0.964196
**IFIT3**	1.586786	12.02167	0.270201	0.695049
**IFIT5**	-1.33536	15.00389	0.048498	0.440385
**IFITM3**	1.447143	8.765556	0.170467	0.557892
**IFNG**	2.52	14.09	0.299088	0.717812
**IL10**	-0.31393	8.318333	0.579123	1
**IL12A**	-1.33107	9.732222	0.182704	0.584654
**IL12B**	-2.80036	17.01444	0.080489	0.440385
**IL13**	0.48	13.68833	0.802984	1
**IL15**	-0.04893	14.39944	0.980214	1
**IL17A**	3.025714	12.05333	0.199182	0.597547
**IL1B**	1.790714	14.92278	0.293799	0.717068
**IL2**	0.289286	13.99	0.845265	1
**IL22RA1**	0.466071	15.955	0.765385	1
**IL23A**	0.008214	11.74389	0.985831	1
**IL2RA**	0.342143	13.10111	0.845144	1
**IL4**	-1.52429	10.30944	0.112287	0.461131
**IL4d2**	-0.6325	8.840556	0.20599	0.605359
**IL5**	3.798929	15.38222	0.100631	0.45284
**IL6**	-2.55714	17.91111	0.088927	0.440385
**IL7R**	0.836786	12.27333	0.223587	0.61917
**IL9**	-0.34929	9.313333	0.803916	1
**INDO**	-2.34429	15.36167	0.084437	0.440385
**IRF7**	0.387143	7.941111	0.599919	1
**KIF1B**	0.115714	7.73	0.623172	1
**LAG3**	0.424286	15.25	0.782852	1
**LTF**	-8.88E-16	7.719	1	1
**LYN**	-2.51	15.42778	0.094805	0.440385
**MARCO**	-0.25536	11.64889	0.270297	0.695049
**MMP9**	1.78E-15	10.176	1	1
**MRC1**	0.8075	8.415556	0.195834	0.597547
**MRC2**	2.8775	13.20056	0.043667	0.440385
**NCAM1**	-0.5175	13.995	0.061554	0.440385
**NEDD4L**	0.477857	16.68167	0.760988	1
**NLRC4**	-0.2275	14.00056	0.717236	1
**NLRP1**	-0.64036	8.024444	0.045192	0.440385
**NLRP10**	-0.01429	10.93389	0.981558	1
**NLRP11**	-8.88E-16	7.64	1	1
**NLRP12**	1.680714	8.947222	0.107938	0.457149
**NLRP13**	-0.08179	7.828889	0.716634	1

**NLRP2**	0.23	11.29389	0.739207	1
**NLRP3**	-0.10786	13.35111	0.824861	1
**NLRP4**	0.414286	9.772222	0.565176	1
**NLRP6**	-0.16357	12.28278	0.790259	1
**NLRP7**	0.027143	10.73611	0.956388	1
**NOD1**	0.08	11.19722	0.901406	1
**NOD2**	-0.30893	12.69722	0.658765	1
**OAS1**	-1.6025	17.96611	0.044492	0.440385
**OAS2**	0.397857	7.949444	0.599787	1
**OAS3**	0.237857	7.825	0.603973	1
**PRF1**	1.777857	9.022778	0.264589	0.695049
**PTPRCv1**	0.451786	12.72889	0.796615	1
**PTPRCv2**	0.128929	13.99278	0.941668	1
**RAB13**	1.78E-15	10.899	1	1
**RAB24**	-1.78E-15	13.366	1	1
**RAB33A**	-0.87821	11.58944	0.09209	0.440385
**RORC**	0.507857	13.835	0.759758	1
**SEC14L1**	-0.44571	12.32333	0.091125	0.440385
**SLAMF7**	-2.52607	17.54778	0.084535	0.440385
**SOCS1**	-2.23929	22.40333	0.15723	0.552224
**SPP1**	0	12.937	1	1
**STAT1**	2.59	9.654444	0.115283	0.461131
**STAT2**	0.592143	8.100556	0.442393	0.964196
**TAGAP**	-0.46893	8.162778	0.396327	0.90589
**TAP1**	1.947143	9.154444	0.12662	0.467518
**TAP2**	0.262857	7.844444	0.602806	1
**TBC1D7**	-8.88E-16	7.64	1	1
**TBX21**	0.563214	15.49056	0.7205	1
**TGFB1**	0	9.92	1	1
**TGFBR2**	-8.88E-16	7.64	1	1
**TIMP2**	0.808571	11.97389	0.15707	0.552224
**TLR1**	-1.17143	8.873889	0.065672	0.440385
**TLR10**	0.368929	9.594444	0.598432	1
**TLR2**	0.065714	12.40111	0.901995	1
**TLR3**	0.135714	12.08056	0.794968	1
**TLR4**	0.007857	12.34111	0.987262	1
**TLR5**	0.065	15.77556	0.895983	1
**TLR6**	0.162143	7.766111	0.491324	1
**TLR7**	0.121786	11.16722	0.824866	1
**TLR8**	0.031071	13.66667	0.956645	1
**TLR9**	0.430714	8.395	0.495496	1
**TNF**	-0.71964	12.27278	0.0918	0.440385
**TNFRSF18**	-0.88964	10.54056	0.12527	0.467518
**TNFRSF1A**	-1.78E-15	10.856	1	1
**TNFRSF1B**	0	10.208	1	1
**TNIP1**	5.219286	11.69944	0.021373	0.440385
**TWIST1**	0.065	7.690556	0.665491	1
**VEGF**	-2.4575	13.66611	0.064223	0.440385
**ZNF331**	-0.26964	11.65278	0.561627	1
**ZNF532**	-0.03607	13.53944	0.983176	1
