# Supplementary material for: Comparative gene expression pattern of immune-related genes using dual-color RT-MLPA in the lesions of cutaneous leishmaniasis caused by L. major and L. tropica
Source: PLoS Negl Trop Dis. 2025 Mar 18;19(3):e0012812. doi: 10.1371/journal.pntd.0012812 (PMC11918365; doi:10.1371/journal.pntd.0012812)
Supplement: S3 Table — Red highlighted genes represent significant DEGs. (PDF) [file pntd.0012812.s004.pdf]

S3 Table. The Limma package was applied to evaluate the DEGs for Iran CL patients relative to controls. Red highlighted genes represents significant DEGs.

Gene name	Log2FC	Average expression	*p*. value	Adj. *p*. value
AIRE	0.203125	10.44273	0.832714	0.935019
AREG	-0.10813	9.196364	0.739628	0.918159
ASAP1	-0.73417	11.63273	0.491885	0.775955
BCL2	0.285	8.242273	0.591213	0.831449
BLR1	-0.23938	12.09091	0.451416	0.75352
BMP6	-1.46729	13.94455	0.299012	0.669333
BPI	0.288979	11.9185	0.479531	0.771484
CAMTA1	-0.46354	9.074545	0.594717	0.831449
CASP8	1.82375	9.491364	0.021629	0.23958
CCL11	0.413125	12.16545	0.799597	0.930861
CCL13	-0.11069	9.7145	0.78383	0.930792
CCL19	0.68375	13.06227	0.003202	0.053516
CCL2	0.324375	7.875909	0.341882	0.683764
CCL22	-0.14854	12.90364	0.719161	0.900515
CCL3	2.02875	9.115455	0.001231	0.02954
CCL4	0.475062	11.7905	0.16136	0.618914
CCL5	1.092708	10.96136	0.172869	0.62233
CCR7	-0.15458	14.94091	0.874518	0.946847
CD14	2.471458	10.91909	0.003345	0.053516
CD163	0.304104	12.6845	0.62027	0.850657
CD19	0.393937	9.1715	0.279141	0.669333
CD209	0.7075	8.509545	0.167623	0.618914
CD274	-2.35125	8.55	0.035642	0.270131
CD3E	-0.08204	9.752	0.820968	0.930861
CD4	0.791458	12.58227	6.26E-06	0.000901
CD8A	0.258333	12.42955	0.747417	0.919898
CLEC7A	1.417292	9.149091	0.021203	0.23958
CTLA4	-0.15563	14.07682	0.495749	0.775955
CX3CL1	-1.08979	10.92409	0.369493	0.700091
CXCL10	0.816042	13.61182	0.000258	0.012382
CXCL13	-0.80875	12.69182	0.501384	0.776336
CXCL9	-1.33104	15.81864	0.323601	0.679798
DSE	-4.44E-15	7.64	1	1
EGF	-1.42292	13.26182	0.304441	0.669333
FASLG	-0.26469	10.4525	0.5184	0.785785
FCGR1A	0.192042	12.533	0.60225	0.833885
FLCN1	-0.19563	10.02773	0.417551	0.735273
FOXP3	0.110208	9.721818	0.89915	0.953153
FPR1	0.191458	10.43091	0.850287	0.939186
GATA3	-1.20792	12.51318	0.221622	0.651298
GBP1	2.523125	9.475	0.01481	0.193875
GBP2	3.157083	10.88273	0.023399	0.240677
GBP5	2.469375	9.435909	0.030549	0.255833
GNLY	0.327292	11.95636	0.679503	0.882726
GZMA	2.443542	10.28545	0.04262	0.306865
GZMB	-0.01167	15.76318	0.989899	1
HCK	0.574167	11.64091	0.527805	0.791708

**HPRT**	0.46625	7.979091	0.54507	0.797246
**IFI16**	-0.12208	14.83455	0.818457	0.930861
**IFI35**	-6.25583	13.60364	0.002336	0.048054
**IFI44**	1.597292	9.7	0.094078	0.457528
**IFI44L**	-0.83875	12.34	0.16541	0.618914
**IFI6**	2.5125	9.467273	0.029476	0.255833
**IFIH1**	-1.23833	14.97773	0.067716	0.417732
**IFIT2**	0.545625	8.036818	0.267926	0.669333
**IFIT3**	1.968125	10.59136	0.058016	0.379742
**IFIT5**	-1.06229	13.78409	0.127115	0.586767
**IFITM3**	2.72375	9.620909	0.0008	0.023051
**IFNG**	2.25	10.41136	0.095318	0.457528
**IL10**	-0.09875	7.853182	0.687897	0.882726
**IL12A**	0.473542	10.03773	0.540848	0.797246
**IL12B**	-1.47125	14.195	0.29293	0.669333
**IL13**	0.82125	10.99727	0.428909	0.735273
**IL15**	0.229167	11.71	0.837622	0.935019
**IL17A**	1.15875	9.047727	0.306029	0.669333
**IL1B**	1.158958	11.88455	0.363913	0.698713
**IL2**	-0.34646	11.54636	0.694166	0.882726
**IL22RA1**	-0.12042	13.27409	0.9002	0.953153
**IL23A**	-0.14646	11.20682	0.651829	0.869106
**IL2RA**	0.841458	10.71364	0.337567	0.683764
**IL4**	1.91625	9.548636	0.004458	0.064202
**IL4d2**	0.074792	8.152727	0.814366	0.930861
**IL5**	0.896875	11.95227	0.511455	0.783506
**IL6**	-1.15333	15.18955	0.362298	0.698713
**IL7R**	-0.69042	11.85455	0.306778	0.669333
**IL9**	0.596667	8.282273	0.322382	0.679798
**INDO**	-0.55375	12.74227	0.627372	0.852279
**IRF7**	-4.44E-15	7.64	1	1
**KIF1B**	0.292292	8.335909	0.560573	0.807225
**LAG3**	0.094583	12.71545	0.911828	0.958417
**LTF**	-0.07219	7.6625	0.282855	0.669333
**LYN**	-0.8525	12.905	0.460484	0.75352
**MARCO**	0.294167	11.23227	0.212162	0.64447
**MMP9**	4.03425	10.574	2.53E-05	0.001821
**MRC1**	-0.50771	9.264091	0.341587	0.683764
**MRC2**	1.338958	12.81045	0.198672	0.635752
**NCAM1**	-0.22458	13.53	0.304489	0.669333
**NEDD4L**	-0.12188	13.99136	0.896369	0.953153
**NLRC4**	-0.25354	13.45227	0.442947	0.750405
**NLRP1**	-0.44833	8.352273	0.138542	0.586767
**NLRP10**	-0.28167	10.35682	0.405557	0.735273
**NLRP11**	-4.44E-15	7.64	1	1
**NLRP12**	0.37125	8.46	0.548106	0.797246
**NLRP13**	0.08875	7.859545	0.592493	0.831449
**NLRP2**	-0.38958	10.42	0.428046	0.735273
**NLRP3**	-0.40229	12.71409	0.244771	0.669333
**NLRP4**	0.14375	9.349545	0.770162	0.930792
**NLRP6**	-0.17	11.70636	0.650849	0.869106

**NLRP7**	-0.24292	10.2	0.456768	0.75352
**NOD1**	-0.46771	10.61318	0.258788	0.669333
**NOD2**	-0.51146	12.01636	0.267018	0.669333
**OAS1**	-1.0625	16.88727	0.137341	0.586767
**OAS2**	0.646875	8.110455	0.192228	0.635752
**OAS3**	0.668125	8.125909	0.188565	0.635752
**PRF1**	1.403542	9.629091	0.229517	0.66101
**PTPRCv1**	1.099583	10.52636	0.20969	0.64447
**PTPRCv2**	0.697708	12.17409	0.325737	0.679798
**RAB13**	-0.4235	10.367	0.410956	0.735273
**RAB24**	-0.15881	12.6645	0.698825	0.882726
**RAB33A**	0.418333	11.12591	0.214823	0.64447
**RORC**	0.312708	10.92909	0.768149	0.930792
**SEC14L1**	0.258333	12.02955	0.137297	0.586767
**SLAMF7**	-0.83875	15.02	0.482177	0.771484
**SOCS1**	-0.89938	21.87591	0.154197	0.618914
**SPP1**	0.957687	12.4915	0.03022	0.255833
**STAT1**	2.421458	12.24773	0.075424	0.417732
**STAT2**	1.575625	8.785909	0.031979	0.255833
**TAGAP**	0.865208	8.460909	0.055989	0.379742
**TAP1**	3.319375	10.05409	0.000735	0.023051
**TAP2**	0.36375	7.904545	0.271524	0.669333
**TBC1D7**	-4.44E-15	7.64	1	1
**TBX21**	-0.16854	12.75909	0.860921	0.939186
**TGFB1**	0.6105	9.054	0.082258	0.438708
**TGFBR2**	0.495	8	0.403871	0.735273
**TIMP2**	-0.02271	13.07182	0.963807	1
**TLR1**	-0.58313	8.650909	0.179383	0.630027
**TLR10**	0.110208	8.546818	0.818841	0.930861
**TLR2**	-0.2075	11.92409	0.42564	0.735273
**TLR3**	-0.31208	11.51636	0.282192	0.669333
**TLR4**	-0.50417	11.655	0.197583	0.635752
**TLR5**	-0.32583	15.08136	0.287798	0.669333
**TLR6**	0.061875	7.9	0.788588	0.930792
**TLR7**	0.058542	10.59091	0.856831	0.939186
**TLR8**	-0.24854	13.12091	0.416668	0.735273
**TLR9**	0.525	8.021818	0.073848	0.417732
**TNF**	0.113125	12.08727	0.692574	0.882726
**TNFRSF18**	0.248125	10.36545	0.288495	0.669333
**TNFRSF1A**	0.209917	10.411	0.688757	0.882726
**TNFRSF1B**	0.724167	9.58	0.085969	0.442129
**TNIP1**	2.474583	10.33136	0.071258	0.417732
**TWIST1**	-4.44E-15	7.64	1	1
**VEGF**	-1.04063	11.10818	0.356023	0.698713
**ZNF331**	-0.43083	11.15	0.157604	0.618914
**ZNF532**	-0.26208	11.27273	0.780993	0.930792
