# Supplementary material for: Comparative gene expression pattern of immune-related genes using dual-color RT-MLPA in the lesions of cutaneous leishmaniasis caused by L. major and L. tropica
Source: PLoS Negl Trop Dis. 2025 Mar 18;19(3):e0012812. doi: 10.1371/journal.pntd.0012812 (PMC11918365; doi:10.1371/journal.pntd.0012812)
Supplement: S4 Table — (PDF) [file pntd.0012812.s005.pdf]

S4 Table. Pearson correlation (*r*) between the significant gene expression and clinical data for Morocco CL patients, and associated *p* values.

**Genes**	**Illness duration**		**Lesion size**		**Age**	
**Correlation**	***r***	***p***	***r***	***p***	***r***	***p***
**CD14**	-0.23483	0.439948	-0.3324	0.421136881	0.126703	0.666014
**CTLA4**	0.233276	0.443072	0.403146	0.322009676	-0.0399	0.892266
**CX3CL1**	-0.37759	0.203381	0.560866	0.148103622	0.349164	0.221095
**CXCL13**	0.350673	0.240096	-0.17075	0.686013858	-0.52747	0.052575
**IFI6**	0.089609	0.77096	0.242872	0.562205921	0.354669	0.213401
**IFIT5**	0.158552	0.604907	0.246623	0.555990629	0.028673	0.922487
**MRC2**	-0.37273	0.209741	0.141646	0.737944698	0.186444	0.523342
**NLRP1**	-0.23025	0.449182	-0.15119	0.720816619	-0.16389	0.575587
**OAS1**	0.113731	0.711422	0.293103	0.481096378	0.294702	0.306395
**TNIP1**	0.107922	0.725635	0.430148	0.287436584	-0.09221	0.753896
**Illness duration**	NA	NA	-0.05286	0.910385008	-0.17889	0.558718
**Lesion size**	-0.05286	0.910385	NA	NA	0.488135	0.219742
**Age**	-0.17889	0.558718	0.488135	0.219742171	NA	NA
