# Supplementary material for: Comparative gene expression pattern of immune-related genes using dual-color RT-MLPA in the lesions of cutaneous leishmaniasis caused by L. major and L. tropica
Source: PLoS Negl Trop Dis. 2025 Mar 18;19(3):e0012812. doi: 10.1371/journal.pntd.0012812 (PMC11918365; doi:10.1371/journal.pntd.0012812)
Supplement: S5 Table — (PDF) [file pntd.0012812.s006.pdf]

**S5 Table.** Pearson correlation (*r*) between the significant gene expression and clinical data for Iran CL patients, and associated *p* values.

| Genes | Illness duration | | Lesion size | | Age | |
|---|---|---|---|---|---|---|
| **Correlation** | ***r*** | ***p*** | ***r*** | ***p*** | ***r*** | ***p*** |
| **CD4** | 0.44646962 | 0.082991251 | -0.018335585 | 0.946264886 | 0.180023764 | 0.504665685 |
| **MMP9** | 0.314254377 | 0.235867183 | -0.011732204 | 0.965603405 | 0.380107426 | 0.146425762 |
| **CXCL10** | 0.089353979 | 0.742093593 | 0.078226244 | 0.773374398 | 0.159136436 | 0.556074301 |
| **TAP1** | 0.226948873 | 0.397960933 | 0.021722541 | 0.936356179 | 0.435183157 | 0.092044649 |
| **IFITM3** | 0.202820836 | 0.451235488 | -0.048742874 | 0.857732629 | 0.452339339 | 0.078544727 |
| **CCL3** | 0.109030255 | 0.687723945 | -0.157201283 | 0.560949039 | 0.097075984 | 0.720603762 |
| **IFI35** | 0.380126152 | 0.146404231 | -0.094765587 | 0.727013715 | 0.173945309 | 0.519392726 |
| **CCL19** | 0.223544906 | 0.405264939 | 0.179342017 | 0.506307639 | 0.115045326 | 0.671371852 |
| **CD14** | 0.44646962 | 0.082991251 | -0.018335585 | 0.946264886 | 0.180023764 | 0.504665685 |
| **IL4** | 0.065283544 | 0.810169877 | -0.23557599 | 0.379768699 | 0.096242104 | 0.722915289 |
| **GBP1** | 0.425702109 | 0.100176507 | -0.114239471 | 0.673554772 | 0.134622482 | 0.619131377 |
| **CLEC7A** | 0.067968482 | 0.802503409 | -0.228483632 | 0.394690956 | 0.350159609 | 0.183655208 |
| **CASP8** | 0.161393883 | 0.550411013 | 0.25808542 | 0.33450195 | 0.513571775 | 0.04187301 |
| **GBP2** | 0.432986997 | 0.093884902 | 0.100081932 | 0.712290168 | 0.282894043 | 0.28838686 |
| **IFI6** | 0.474932899 | 0.063026532 | -0.284432698 | 0.285659099 | 0.101796436 | 0.707561839 |
| **SPP1** | 0.282158726 | 0.289695932 | -0.181685946 | 0.500672882 | 0.081309234 | 0.764672934 |
| **GBP5** | 0.659317681 | 0.005463966 | -0.117999971 | 0.663389393 | 0.18469479 | 0.493483251 |
| **STAT2** | 0.245707251 | 0.358997165 | 0.189290424 | 0.48259764 | 0.039853113 | 0.883496448 |
| **GZMA** | -0.07536407 | 0.781475365 | -0.170377062 | 0.528128614 | 0.104078344 | 0.70128423 |
| **lesion duration** | 1 | 0 | 0.234953978 | 0.381064926 | 0.336108969 | 0.203088389 |
| **Lesion size** | 0.234953978 | 0.381064926 | 1 | 0 | 0.018264467 | 0.946473027 |
| **Age** | 0.336108969 | 0.203088389 | 0.018264467 | 0.946473027 | 1 | 0 |
